# Grave Crisis for Poor-Law Nurses

**Published:** 1916-08-19

**Authors:** Tom Percival

**Affiliations:** *President*, National Poor-Law Officers' Association (Incorporated). Guardians' Hall, North Shields


### NATIONAL POOR-LAW OFFICERS' ASSOCIATION.

### GRAVE CRISIS FOR POOR-LAW NURSES.

*To the Editor of* THE HOSPITAL.

SIR,—I am rather surprised that you should have been at the trouble to belabour this Association at such length in your issue of the 12th inst. for what is obviously a slip. You state that Mr. Dawes, at a recent meeting, is reported to have said that 75 per cent. of the trained nurses in this country were Poor-Law trained, whereas his reference was, of course, to the number of *beds*. The figures he quoted were taken from the Local Government Board Return on Hospital Accommodation; their accuracy has not been impugned so far as I am aware, and they show that approximately 75 per cent. of the hospital beds provided for the sick in this country are in the infirmaries of Poor-Law institutions.

The importance of the stake which Poor-Law nurses have in the question of the College of Nursing, or, indeed, any question affecting the nursing profession, needs no exaggeration to emphasise their claim for due consideration.

But why this attitude of unfriendly criticism? This Association represents the Poor-Law service as a whole, and has always included a considerable proportion of medical officers and nurses. We are now making efforts (and successful efforts) to organise Poor-Law nurses systematically throughout the country, interesting the nurses in the question of the College of Nursing, and assisting them to establish a separate nursing section with its own officers and machinery so as to enable them to voice their needs—in fact, carrying out practically your own suggestion as to what is needed for their benefit.

Is it unreasonable to hold that this task is one in which we might properly expect you to help instead of hinder?—Yours faithfully,

TOM PERCIVAL, *President*,
National Poor-Law Officers' Association
(Incorporated).

Guardians' Hall, North Shields, August 15, 1916.

[We hope the President of the above Association will dismiss from his mind the idea of any unfriendly criticism on our part. The points in question are of the first importance to the future of Poor-Law nursing and to the best interests of the nursing profession. First, then, Mr. Percival will perceive, on referring to the second sentence in his letter, that Mr. Dawes could not have meant beds, for he used the words "Poor-Law trained." Again, the Local Government Board Return on Hospital Accommodation does not warrant the statement that 75 per cent. of the hospital beds in this country are Poor-Law. An examination of the return reveals the fact that the Poor-Law infirmaries where presumably fully-trained nurses are employed, including those to which training schools are attached, contain altogether 32,069 beds, which total includes 406 beds in two infirmaries each classed A and C—*i.e.*, where there are beds in a separate infirmary and beds in sick wards in the workhouse. It thus appears that the actual hospital beds, properly so-called, under the Poor Law do not probably exceed 40,000 as an outside figure. Eight hundred and thirty-two voluntary hospitals of which particulars are available contain 54,311 beds, of which 42,115, or 77 per cent., are on an average in daily occupation throughout the year. These figures abundantly justify our criticisms of the original statement, and demonstrate that, whether Mr. Dawes referred to nurses or to beds, the contention advanced was absurd on the face of it.

While on the question of Poor-Law hospital accommodation it may be well to point out that the total of 94,001 beds shown in the return under the heading of Poor-Law infirmaries is misleading. When the figures are examined in detail the return reveals that in addition to the actual number of beds in Poor-Law infirmaries (32,069) the compilers of the return have included the beds in (*a*) buildings set apart for the accommodation of sick persons under the control of the workhouses, and (*b*) sick wards in workhouse buildings. (*a*) and (*b*) must include the infirm, chronic, senile, imbecile, and others. These ought not to have been included as hospital beds nor as beds in Poor-Law infirmaries in the Return of Hospital Accommodation, as we have shown.

A GRAVE CRISIS IN POOR-LAW NURSING.

We agree that all fully-trained nurses have important interests at stake in the organisation of the College of Nursing. Further, it is because we hold that every question affecting the nursing profession claims the utmost consideration, that we agree with the majority of fully-trained Poor-Law nurses and their superintendents that the organisation of Poor-Law nurses should be undertaken by the nurses themselves and their leaders, and not by the National Poor-Law Officers' Association, which has other highly important and extensive interests to look after and defend. We are aware that the Poor-Law Officers' Association have proposed that superintendent-nurses and matrons of Poor-Law infirmaries should co-operate with the officers of the Association to induce Poor-Law nurses to become members of it. There is no doubt, however, that there exists an indifference on the part of the nursing profession to the Poor-Law Officers' Association which is not confined to any special district or part of the country. This indifference is due partly to the fact that very few nurses join the Poor-Law Nursing Service with the intention of remaining in it for any length of time, and they take little interest, therefore, in questions of Poor-Law administration and organisation. In fact very few of the matrons, superintendent-nurses, and sisters who have definitely chosen the Poor-Law Service as a career have joined the Association. They, on the contrary, would like to see the sick removed from the workhouse into separate infirmaries, the control of the nursing staff removed from the hands of the workhouse master, and the association of the Poor-Law infirmary with the Guardians abolished. The National Poor-Law Officers' Association, on the other hand, stands for the maintenance of the present system of Poor-Law administration, and the reforms it advocates are limited by this consideration. The fundamental differences between these two views cannot be reconciled. Believing as we do that the Poor-Law infirmaries should develop into State hospitals, and that defects in them are the result of their control by a number of small Boards of Guardians, acting independently of one another, and elected for any reason except their knowledge of hospital work, we cannot urge Poor-Law nurses to join the National Association. We are of opinion that Poor-Law nurses will be well advised to form an independent Association of their own, that is their own society confined to nurses, to increase their interest in the Poor-Law Nursing Service as a whole, and to bring their collective influence and experience to bear upon the solution of pressing problems of which the College of Nursing and State Registration are at the moment the most prominent.—Ed. The Hospital.]

